# Supplementary material for: Identification of photoperiod-induced specific miRNAs in the adrenal glands of Sunite sheep (Ovis aries)
Source: Front Vet Sci. 2022 Jul 22;9:888207. doi: 10.3389/fvets.2022.888207 (PMC9354845; doi:10.3389/fvets.2022.888207)
Supplement: Supplementary file 1 [file Data_Sheet_1.ZIP › Supplementary file/miRDeep2 file.zip!/miRDeep2 file/Know_Structure/SPLP42A2/oar-mir-1197.pdf]

miRBase precursor : oar-mir-1197
Total read count : 69
oar-miR-1197-5p read count : 3
oar-miR-1197-3p read count : 60
remaining reads : 6

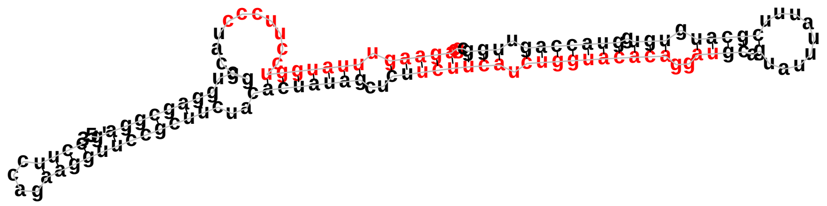

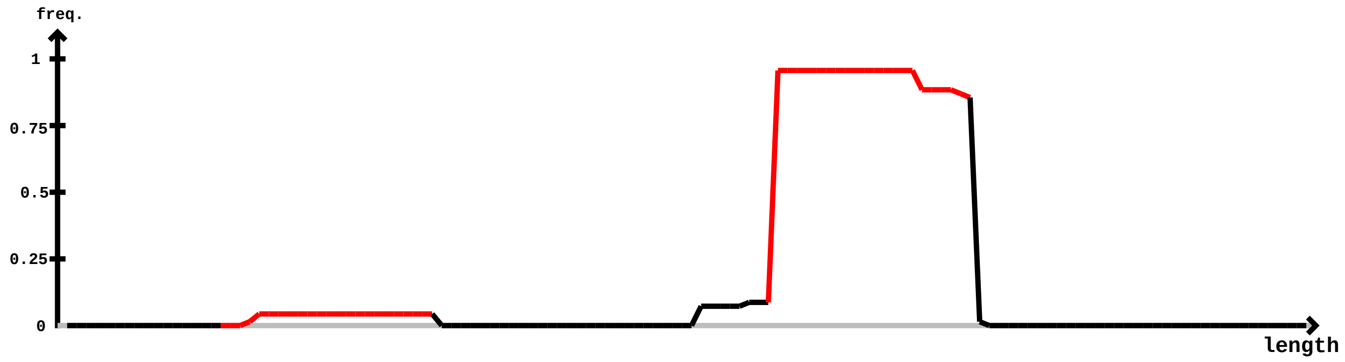


```
                                                                              oar-miR-1197-3p
                   oar-miR-1197-5p
5'- gaggcgaggucggcaucccuuccugguauuugaagacgcgguugaccaugguguguacgcuuuauuuaugacguaggacacauggucuacuucuucucgauaucacaucuucgccuuggaagaccuucc   -3'    exp
    (((((((((..(...........(((((((.(((((...(((.(((((((.(((.(((((.........).))))..))))))))))).))))))))..))))))))..)))))))))((((....))))    reads      mm
    ..................cuuccugguauuugaagacg...........................................................................................    1          0
    ...................uuccugguauuugaagacg...........................................................................................    2          0
    .................................................................uuaugacguaggacacauggucu.........................................    5          0
    ......................................................................acguaggacacauggucuacuucu...................................    1          0
    .........................................................................uaggacacauggucuacuu.....................................    1          0
    .........................................................................uaggacacauggucuacuuc....................................    1          0
    .........................................................................uaggacacauggucuacuucu...................................    57         0
    .........................................................................uaggacacauggucuacuucuu.................................    1          0
```
